# Validation and minimum important difference of the UCSD Shortness of Breath Questionnaire in fibrotic interstitial lung disease

**DOI:** 10.1186/s12931-021-01790-0

**Published:** 2021-07-08

**Authors:** Tao Chen, Amy Po Yu Tsai, Seo Am Hur, Alyson W. Wong, Mohsen Sadatsafavi, Jolene H. Fisher, Kerri A. Johannson, Deborah Assayag, Julie Morisset, Shane Shapera, Nasreen Khalil, Charlene D. Fell, Helene Manganas, Gerard Cox, Teresa To, Andrea S. Gershon, Nathan Hambly, Andrew J. Halayko, Pearce G. Wilcox, Martin Kolb, Christopher J. Ryerson

**Affiliations:** 1 Department of Medicine, University of British Columbia, Vancouver, BC Canada; 2 Centre for Heart Lung Innovation, St. Paul’s Hospital, Vancouver, BC Canada; 3 Department of Respiratory Medicine, Shanghai Pulmonary Hospital, Tongji University, School of Medicine, Shanghai, China; 4 Respiratory Evaluation Sciences Program, Collaboration for Outcomes Research and Evaluation, Faculty of Pharmaceutical Sciences, The University of British Columbia, Vancouver, BC Canada; 5 Department of Medicine, University of Toronto, Toronto, ON Canada; 6 Department of Medicine, University of Calgary, Calgary, AB Canada; 7 Department of Medicine, McGill University, Montreal, QC Canada; 8 Département de Médecine, Centre Hospitalier de l’Université de Montréal, Montreal, QC Canada; 9 Department of Medicine, Firestone Institute for Respiratory Health, McMaster University, Hamilton, ON Canada; 10 Child Health Evaluative Sciences, The Hospital for Sick Children, Toronto, ON Canada; 11 Dalla Lana School of Public Health, University of Toronto, Toronto, ON Canada; 12 Sunnybrook Health Sciences Centre, Department of Medicine, University of Toronto, Toronto, ON Canada; 13 Departments of Internal Medicine and Physiology and Pathophysiology, University of Manitoba, Winnipeg, MB Canada; 14 Department of Medicine and Centre for Heart Lung Innovation, University of British Columbia, 8B Providence Wing, 1081 Burrard Street, Vancouver, BC V6Z 1Y6 Canada

**Keywords:** Dyspnea, Interstitial lung disease, Pulmonary fibrosis, Minimum clinically important difference

## Abstract

**Rationale:**

The University of California, San Diego Shortness of Breath Questionnaire (UCSDSOBQ) is a frequently used domain-specific dyspnea questionnaire; however, there is little information available regarding its use and minimum important difference (MID) in fibrotic interstitial lung disease (ILD). We aimed to describe the key performance characteristics of the UCSDSOBQ in this population.

**Methods:**

UCSDSOBQ scores and selected anchors were measured in 1933 patients from the prospective multi-center Canadian Registry for Pulmonary Fibrosis. Anchors included the St. George’s Respiratory Questionnaire (SGRQ), European Quality of Life 5 Dimensions 5 Levels questionnaire (EQ-5D-5L) and EQ visual analogue scale (EQ-VAS), percent-predicted forced vital capacity (FVC%), diffusing capacity of the lung for carbon monoxide (DLCO%), and 6-min walk distance (6MWD). Concurrent validity, internal consistency, ceiling and floor effects, and responsiveness were assessed, followed by estimation of the MID by anchor-based (linear regression) and distribution-based methods (standard error of measurement).

**Results:**

The UCSDSOBQ had a high level of internal consistency (Cronbach’s alpha = 0.97), no obvious floor or ceiling effect, strong correlations with SGRQ, EQ-5D-5L, and EQ-VAS (|r| > 0.5), and moderate correlations with FVC%, DLCO%, and 6MWD (0.3 < |r| < 0.5). The MID estimate for UCSDSOBQ was 5 points (1–8) for the anchor-based method, and 4.5 points for the distribution-based method.

**Conclusion:**

This study demonstrates the validity of UCSDSOBQ in a large and heterogeneous population of patients with fibrotic ILD, and provides a robust MID estimate of 5–8 points.

**Supplementary Information:**

The online version contains supplementary material available at 10.1186/s12931-021-01790-0.

## Background

Fibrotic interstitial lung diseases (ILDs) are a heterogeneous group of diseases with different etiologies. Some patients experience progressive decline in lung function and worsening dyspnea. Available treatments slow the decline of pulmonary function but do not cure or even stop the disease from progressing [1–4], and few treatments have a measurable effect on patient-reported outcomes. There is therefore an urgent need to develop and test new therapies, and particularly those focusing on improving quality of life and alleviating common symptoms of ILD such as dyspnea. Many health-related quality of life questionnaires have been validated in patients with ILD, including disease-specific instruments such as the King’s Brief Interstitial Lung Disease questionnaire (K-BILD) [5], and generic instruments such as European Quality of Life 5 Dimensions 5 Levels questionnaire (EQ-5D-5L) [6], and European Quality of Life visual analogue scale (EQ-VAS) [6]; however, there are limited data on how to best measure dyspnea and response to therapy for these patients.

The University of California, San Diego Shortness of Breath Questionnaire (UCSDSOBQ) is a frequently used domain-specific dyspnea questionnaire, focusing on the severity of dyspnea during common daily activities. The UCSDSOBQ has been validated in patients with COPD [7, 8] and idiopathic pulmonary fibrosis (IPF) [9], including estimation of the minimum important difference (MID) in these populations. However, there is little information available regarding its use in other fibrotic ILDs, which limits the robustness of clinical trials that are needed in these patients. The goal of this study was therefore to describe the key performance characteristics of the UCSDSOBQ in patients with fibrotic ILD of any etiology, with specific objectives to validate the UCSDSOBQ in this population and quantify its MID.

## Methods

### Study population

The Canadian Registry for Pulmonary Fibrosis (CARE-PF) is a multi-center prospective registry that enrols adults with any type of fibrotic ILD who provide informed consent and are able to complete questionnaires in English or French [10, 11]. All patients were enrolled between January 2015 and March 2020. ILD diagnoses are made by multidisciplinary discussion at each center using established diagnostic criteria where available [12]. IPF was defined according to recent guideline criteria [13]. Probable IPF was defined as a diagnosis of IPF by multidisciplinary discussion without meeting guideline criteria. Given the absence of diagnostic guidelines at the time of enrolment, fibrotic hypersensitivity pneumonitis (HP) was defined by HP being the most likely diagnosis from at least two of three domains (exposure history, imaging findings, and bronchoscopic/pathological findings). Connective tissue disease-associated ILD (CTD-ILD) was diagnosed in collaboration with a rheumatologist using diagnostic criteria where available. Patients were considered to have unclassifiable ILD when a confident diagnosis was unable to be made after multidisciplinary discussion [14]. Approval for this sub-study was obtained from the research ethics boards of each CARE-PF center (coordinating center: Providence Health Care and University of British Columbia Research Ethics Board #H18-00993).

### Data collection

Demographic data and smoking history were collected at the baseline study visit using standardized patient-completed questionnaires, which also included assessment of dyspnea and patient-reported anchors as described below. The baseline visit was the one closest to the diagnosis, which was limited to being ≤ 6 months.

#### UCSD Shortness of Breath Questionnaire

The UCSDSOBQ is a 24-item patient-completed survey that evaluates the severity of dyspnea during common activities [15]. Each question is scored from 0 (“not at all”) to 5 (“unable to do because of breathlessness”), with the sum of all scores representing the overall severity of the breathlessness on a scale of 0–120. The UCSDSOBQ was derived primarily in patients with COPD, cystic fibrosis, and post lung-transplantation [15], and has since been used as a key patient-reported outcome measure in clinical trials for a variety of respiratory diseases, including IPF [1, 3, 16–18]. The questionnaire can be completed in < 5 min, with a high test–retest reliability of 0.94 in patients with chronic obstructive pulmonary disease (COPD) [19]. The MID for the UCSDSOBQ in patients with COPD is reported at 5 for a small change (range 5–6), 11 for a moderate change (range 9–15), and 16 for a large change (range 14–20) [7, 8]. In patients with IPF, the reported MID of the UCSDSOBQ is 8 (range 5–11) [9], without any reported MID available for other ILD subtypes.

#### Anchors

Pre-selected anchors included patient-reported measurements and both physiological and functional measures of ILD severity. Patient-reported anchors consisted of three health-related quality of life measurements, including the St. George’s Respiratory Questionnaire (SGRQ), EQ-5D-5L, and EQ-VAS. The SGRQ is a disease-specific self-administered questionnaire that measures the impact of lung disease on health-related quality of life. It was developed in patients with COPD and asthma [20], and now is validated in other respiratory diseases, including fibrotic ILD [21]. The total weighted score of the SGRQ ranges from 0 to 100, with higher scores indicating worse health state. Scores are also calculated for three separate domains of symptoms, activity, and impacts. The MID estimate of the SGRQ total score ranges from 5 to 8 derived from a previous study of patients with IPF [21]. The EQ-5D-5L is a patient-completed generic health-related quality of life instrument that includes five dimensions: mobility, self-care, usual activities, pain/discomfort, and anxiety/depression. Each dimension ranges from 1 (“no problems”) to 5 (“extreme problems”), with these scores then converted into an index score using an algorithm that was previously derived from a representative Canadian adult population [22]. The EQ-VAS records self-rated health on a visual analogue scale ranging from 0 to 100. For both the EQ-5D-5L and EQ-VAS, a higher score represents better health state. We have previously calculated the MID for the EQ-5D-5L and EQ-VAS to be 0.054 and 5 in patients with fibrotic ILD, respectively [6]. Objective measures of ILD severity consisted of two pulmonary function measures, including percent-predicted forced vital capacity (FVC%) and diffusing capacity of the lung for carbon monoxide (DLCO%), as well as the 6-min walk distance (6MWD) as a measure of functional capacity. FVC%, DLCO%, and 6MWD were recorded within 3 months of completion of the UCSDSOBQ, with 84% of tests completed within one month. Previously suggested MID ranges for FVC% and 6MWD are 2–6% and 20.7–35.4 m, respectively, which are derived from patients with IPF [23, 24], while the estimated MID of the DLCO% is 11% as determined in patients with COPD [25].

### Statistical analysis

Data are presented as mean ± standard deviation, number (percent), or median (interquartile range). P < 0.05 was used to indicate statistical significance. All analyses were performed using Stata version 16 (StataCorp LLC).

#### Internal consistency of the UCSDSOBQ

Internal consistency of the UCSDSOBQ at the baseline study visit was tested using Cronbach’s alpha that indicates the average inter-correlation among items. The value of Cronbach’s alpha can range from 0 to 1, with 0.70 to 0.95 generally considered to represent acceptable internal consistency [26].

#### Concurrent validity of the UCSDSOBQ

The concurrent validity of the UCSDSOBQ was tested using a Spearman correlation between the UCSDSOBQ and each available anchor measured at baseline. The strength of association was assessed by the absolute Spearman’s correlation coefficient (|r|), with |r| > 0.5 representing strong correlation, 0.3 < |r| < 0.5 indicating moderate correlation, and 0.1 < |r| < 0.3 indicating weak association [27].

#### Responsiveness of the UCSDSOBQ

Patients with longitudinal data available at 6 months (allowable range 4–8 months with use of the measurement closest to 6 months if multiple measurements available) for both the UCSDSOBQ and at least one anchor were divided into three tertiles based on the magnitude of change in UCSDSOBQ. Across the tertiles of change in UCSDSOBQ, the mean change of each anchor was calculated to indicate the responsiveness of the UCSDSOBQ, with a greater difference across tertiles indicating better responsiveness. A secondary analysis considered longitudinal data available at 12 months (allowable range 9–15 months).

#### Calculation of MID for the UCSDSOBQ

Anchor-based estimation of the UCSDSOBQ MID was based on a series of linear regression models with the UCSDSOBQ as the outcome variable and each anchor as the sole predictor variable, limiting this analysis to anchors with |r| > 0.3. The MID of each pre-selected anchor was entered into the linear regression equation in order to determine the corresponding MID for the UCSDSOBQ. A range for the UCSDSOBQ MID was generated for anchors that had a MID range previously specified, while a single value was generated for anchors that had a single MID value. The normal distribution of residuals was the standard to verify the validation of all linear regression models. Interpretation was made with and without consideration of MID estimates obtained using the EQ-5D-5L and EQ-VAS since we had previously calculated these MID values in a subgroup of patients reported in this analysis [6]. Distribution-based estimation of the MID was calculated based on the standard error measurement (SEM) method ($SEM = \sigma _{x} \sqrt {1 - r_{{xx}} }$; *σ*_*x*_ standard derivation, *r*_*xx*_ reliability coefficient) [28].

#### Association between the UCSDSOBQ and mortality

Two separate Cox proportional hazard analyses were used to determine the association of both baseline UCSDSOBQ and 6-month change in UCSDSOBQ with subsequent mortality. A Kaplan–Meier curve and two-sided log-rank test were used to display and compare survival for the three groups: decreased UCSDSOBQ score more than the average MID estimate, increased UCSDSOBQ score more than the average MID estimate, and change of UCSDSOBQ less than the average MID estimate.

## Results

### Baseline characteristics

The total study population consisted of 1933 patients, including 539 (28%) with IPF, 701 (36%) with CTD-ILD, 344 (18%) with unclassifiable ILD, 151 (8%) with HP, and 67 (3%) with other ILDs (Table 1 and Additional file 1: Table E2). The mean age of the cohort was 62 ± 13 years old, and 1056 (55%) were male. Patients had on average mild reduction in FVC% (75 ± 20%) and moderate reduction in DLCO% (58 ± 20%). There were no substantial differences in baseline data for patients with and without six-month follow-up data (Additional file 1: Table E1).

**Table 1:** Baseline characteristics

Variable	Value
Total sample size	1933
IPF	539 (28%)
CTD-ILD	701 (36%)
Unclassifiable ILD	344 (18%)
HP	151 (8%)
Other ILD	198 (10%)
Age, years	62 ± 13
Male sex	1056 (55%)
Ever-smoker	1214 (63%)
Smoking pack-years (IQR)	21 (8–37)
FVC, %-predicted	75 ± 20
DLCO, %-predicted	58 ± 20
6MWD, meters	433 ± 129
UCSDSOBQ total score	37 ± 27
SGRQ total score	41 ± 20
EQ-5D-5L	0.79 ± 0.19
EQ-VAS	69 ± 19

Data shown are number (%), mean ± standard deviation, or median (interquartile range)

*6MWD* 6-min walk distance, *CTD-ILD* connective tissue disease-associated ILD, *DLCO* diffusing capacity of the lung for carbon monoxide, *EQ-5D-5L* European Quality of Life 5 Dimensions 5 Levels questionnaire, *EQ-VAS* European Quality of Life visual analogue scale, *FVC* forced vital capacity, *HP* hypersensitivity pneumonitis, *ILD* interstitial lung disease, *IPF* idiopathic pulmonary fibrosis, *IQR* interquartile range, *SD* standard deviation, *SGRQ* St. George’s Respiratory Questionnaire, *UCSDSOBQ* University of California, San Diego Shortness of Breath Questionnaire

### Internal consistency and concurrent validity of the UCSDSOBQ

**Fig. 1:**
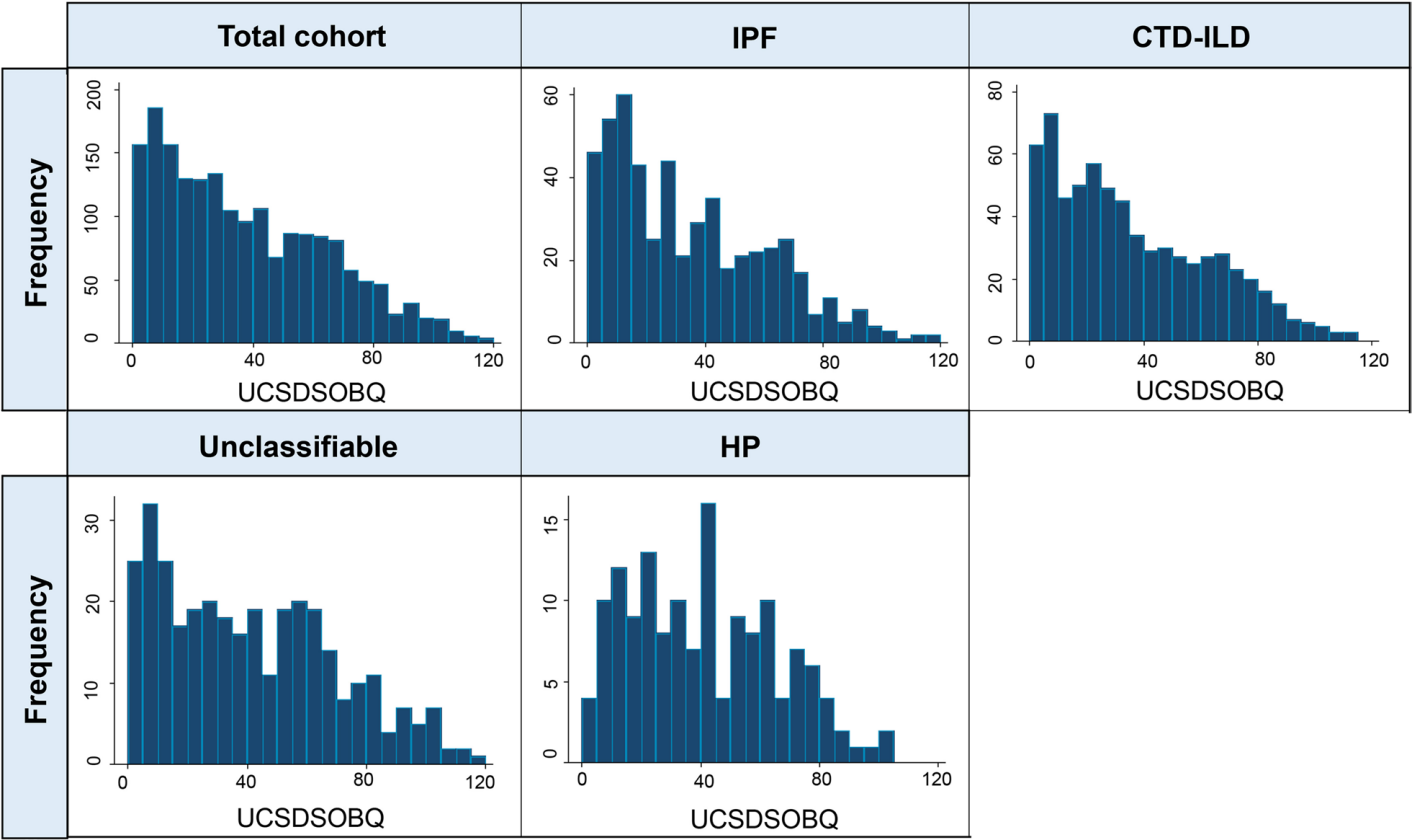
Frequency distribution for the UCSDSOBQ. *CTD-ILD* connective tissue disease-associated ILD; *HP* hypersensitivity pneumonitis; *IPF* idiopathic pulmonary fibrosis; *UCSDSOBQ* University of California, San Diego Shortness of Breath Questionnaire

**Fig. 2:**
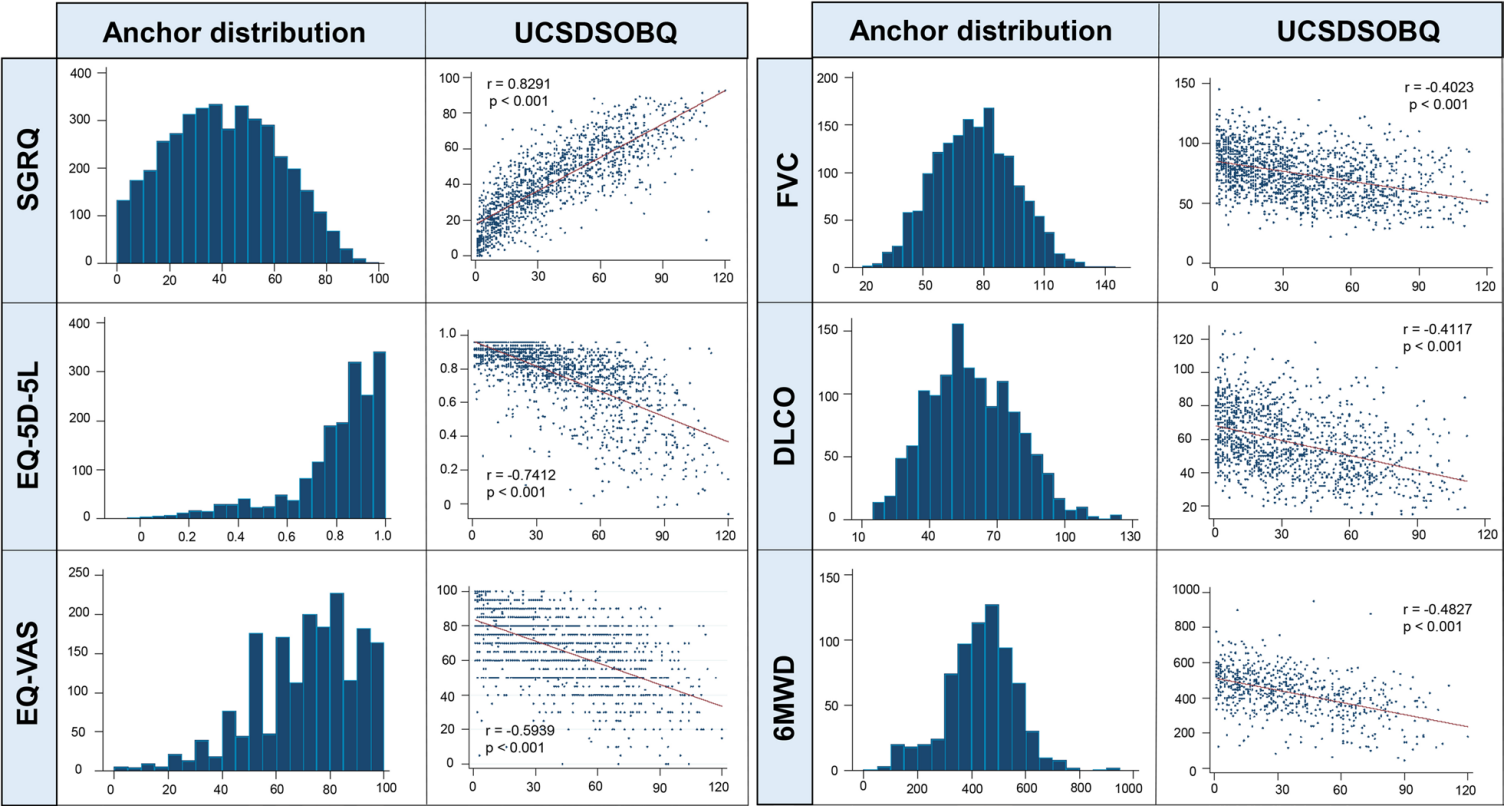
Association of the UCSDSOBQ with selected anchors. *6MWD* 6-min walk distance; *DLCO* diffusing capacity of the lung for carbon monoxide; *EQ-5D-5L* European Quality of Life 5 Dimensions 5 Levels questionnaire; *EQ-VAS* European Quality of Life visual analogue scale; *FVC* forced vital capacity; *SGRQ* St. George’s Respiratory Questionnaire; *UCSDSOBQ* University of California, San Diego Shortness of Breath Questionnaire

Cronbach’s alpha of the UCSDSOBQ was 0.97, indicating a high level of internal consistency. There were 54 (2.8%) patients with a score of 0, and 2 (0.1%) patients with a score of 120, indicating no significant floor or ceiling effect [29]. The UCSDSOBQ scores had a non-normal (right-skewed) distribution, with the majority of patients scoring ≤ 40. A similar finding was observed in each diagnostic category (Fig. 1). All anchors were at least moderately associated with UCSDSOBQ (Fig. 2), suggesting their appropriateness for anchor-based MID estimation. The UCSDSOBQ had strong correlations with patient-reported quality of life questionnaires (|r| > 0.50 for SGRQ, EQ-5D-5L, and EQ-VAS) and moderate correlations with FVC%, DLCO%, and 6MWD (0.3 < |r| < 0.5).

### Responsiveness of the UCSDSOBQ

The responsiveness analysis included 799 patients who had longitudinal UCSDSOBQ scores obtained 6 months after baseline (Table 2). Consistent with the correlation analysis, patient-reported measurements (SGRQ, EQ-5D-5L, and EQ-VAS) demonstrated superior responsiveness, showing clear separation in the change of these anchors across tertiles of change in the UCSDSOBQ. The change of 6MWD, FVC, and DLCO showed only mild differences across these same tertiles. Results were similar when comparing IPF with non-IPF ILD. Results were also similar when repeating this analysis using UCSDSOBQ scores obtained 12 months after baseline (allowable range 9–15 months) (Additional file 1: Table E3).

**Table 2:** Change in anchors across tertiles of change in UCSDSOBQ over 6 months

Tertiles of change in UCSDSOBQ	Number of patients	Tertile 1 (decrease in UCSDSOBQ)	Tertile 2 (minimal change)	Tertile 3 (increase in UCSDSOBQ)
mean ∆UCSDSOBQ	468	− 11.8 ± 9.4	2.3 ± 2.9	20.18 ± 13.71
∆ reported in SGRQ		** − 4.7 ± 10.4**	**0.7 ± 8.4**	**7.5 ± 11.0**
mean ∆UCSDSOBQ	740	− 12.6 ± 10.8	2.2 ± 2.9	20.7 ± 13.4
∆ reported in EQ-5D-5L		**0.037 ± 0.130**	** − 0.006 ± 0.102**	** − 0.084 ± 0.167**
mean ∆UCSDSOBQ	760	− 12.8 ± 11.3	2.2 ± 2.9	20.5 ± 13.4
∆ reported in EQ-VAS		**4.1 ± 15.3**	**0.2 ± 13.4**	** − 7.5 ± 18.9**
mean ∆UCSDSOBQ	555	− 12.4 ± 11.1	2.0 ± 2.9	19.0 ± 12.0
∆ reported in FVC		**1.4 ± 5.9**	** − 1.0 ± 6.5**	** − 1.8 ± 6.5**
mean ∆UCSDSOBQ	442	− 12.4 ± 11.1	2.2 ± 3.0	18.0 ± 10.3
∆ reported in DLCO		**0.4 ± 7.2**	** − 2.0 ± 9.2**	** − 2.0 ± 8.2**
mean ∆UCSDSOBQ	245	− 13.3 ± 10.9	1.9 ± 2.7	18.7 ± 11.8
∆ reported in 6MWD		** − 2.8 ± 54.9**	** − 3.9 ± 91.3**	** − 30.3 ± 101.9**

Data shown in bold font are the change in each anchor across tertiles of change in UCSDSOBQ. Data shown in regular font are the mean change in UCSDSOBQ for each tertile of change, based on the patients with available data for that anchor

*6MWD* 6-min walk distance, *DLCO* diffusing capacity of the lung for carbon monoxide, *EQ-5D-5L* European Quality of Life 5 Dimensions 5 Levels questionnaire, *EQ-VAS* European Quality of Life visual analogue scale, *FVC* forced vital capacity, *SGRQ* St. George’s Respiratory Questionnaire, *UCSDSOBQ* University of California, San Diego Shortness of Breath Questionnaire

### Estimation of the MID for the UCSDSOBQ

The anchor-based MID estimates of the UCSDSOBQ ranged from 1.1 to 8.4, with a mean value of 4.6. There was no difference in MID estimates comparing patients with a baseline UCSDSOBQ above and below the median value. Sub-group analyses identified similar results for MID estimates comparing IPF (mean 4.7; range 1.0–8.5) with non-IPF (4.6; 1.1–8.3), CTD-ILD (4,4; 1.0–8.2) with non-CTD-ILD (4.5; 1.1–8.5), male (4.6; 1.0–8.2) with female (4.3; 1.1–8.5), and younger than the median age (4.4; 1.1–7.9) with older (4.6; 1.1–9.1) (Additional file 1: Tables E4–E7). The distribution-based MID was 4.5 by the SEM approach (Table 3). Results were the same when limited to the 84% of patients who had measurements taken within one month.

**Table 3:** Estimates of MID for UCSDSOBQ by anchor-based and distribution-based methods

Ancho-based MID estimates				
Anchor	Linear regression equation		MID for anchor	MID for UCSDSOBQ
SGRQ	UCSD = -5.524 + 1.047*SGRQ		5–8	5.2–8.4
EQ-5D-5L	UCSD = 115.2 – 100.2*EQ-5D-5L		0.054	5.4
EQ-VAS	UCSD = 94.13 – 0.84*EQ-VAS		5	4.2
FVC%	UCSD = 77.24 – 0.54*FVC%		2–6%	1.1–3.2
DLCO%	UCSD = 65.77 – 0.54*DLCO%		11%	5.9
6MWD	UCSD = 77.41 – 0.097*6MWD		20.7–35.4 m	2.0–3.4
Distribution-based MID estimates				
Standard error measurement approach		4.5		

*6MWD* 6-min walk distance, *DLCO* diffusing capacity of the lung for carbon monoxide, *EQ-5D-5L* European Quality of Life 5 Dimensions 5 Levels questionnaire, *EQ-VAS* European Quality of Life visual analogue scale, *FVC* forced vital capacity, *MID* minimum important difference, *SGRQ* St. George’s Respiratory Questionnaire, *UCSDSOBQ* University of California, San Diego Shortness of Breath Questionnaire

### Association of the UCSDSOBQ with mortality

**Fig. 3:**
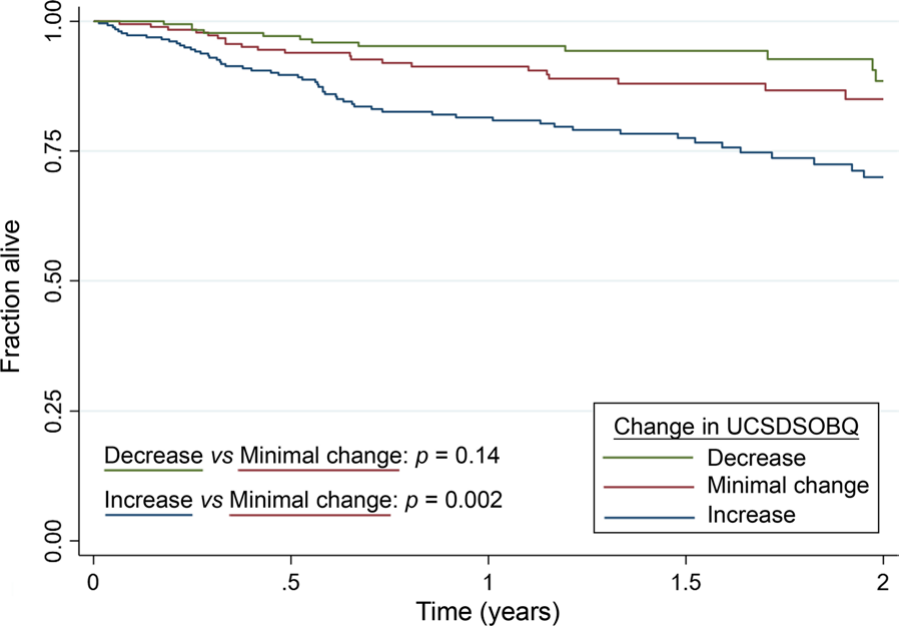
Survival by MID of UCSDSOBQ. Decrease: patients with decreased UCSDSOBQ more than 4.6 at 6 months; Minimal change: patients with the change of UCSDSOBQ less than 4.6 at 6 months; Increase: patients with increased UCSDSOBQ more than 4.6 at 6 months

Baseline UCSDSOBQ was associated with mortality, with a hazard ratio of 1.13 per 5-unit increase (95%CI 1.11–1.16, P < 0.001). The 6-month change of UCSDSOBQ was also associated with mortality, with a hazard ratio of 1.19 per 5-unit change (95%CI 1.09–1.26, P < 0.001) (Fig. 3).

## Discussion

Symptom assessment and management is vital for optimal management of patients with fibrotic ILD given the absence of a cure; however, there are limited data on the best methods to measure the most common ILD symptoms. We show in a large and diverse population of patients with fibrotic ILD that the UCSDSOBQ is easily administered, highly reproducible, and responsive to change, with an estimated MID of 5 to 8 points. These findings suggest that the UCSDSOBQ is appropriate for use as a key outcome in future clinical trials of this patient population, and also has potential utility in routine clinical practice as a robust and precise measure of dyspnea severity. Although the UCSD has been validated in patients with IPF and COPD, we confirmed its appropriateness in a contemporary cohort of patients with IPF and further expanded its application to other subtypes of fibrotic ILD given the varying disease trajectory and prognosis of non-IPF ILD.

We estimated the MID for the UCSDSOBQ through multiple analyses, with the broad range of MID (1.1–8.4 points) dependent on the previously established MID of the selected anchors. For example, we used 2–6% as the MID of FVC% [23], despite the likelihood that the lower limit of this range is probably not appropriate for clinical use. This lower limit of 2% may have some utility when averaged across large populations, but this threshold has limited utility in an individual patient given the substantial measurement error that exceeds 2%. We therefore place less importance on the lower limit of anchor-based estimates and instead prioritize both the mean value of the anchor-based estimation (4.6) and the MID estimate from distribution-based method (4.5). Given the need to use a whole number, we therefore suggest a MID of 5 points in fibrotic ILD, which is consistent with the value previously derived in patients with COPD [7, 8]. This threshold is smaller than the MID (8 points) previously calculated in patients with IPF [9], which can be explained by differences in the anchors used in this previous study as well as the previous study’s primary analysis focusing on the association of changes in UCSDSOBQ and changes in the anchors, which introduces greater variability to these measurements. We further verified the clinical relevance of a 5-point change in UCSDSOBQ by confirming its strong association with mortality. The upper limit of the anchor-based MID (8 points) provides a more conservative choice, representing a change that is of more certain clinical importance.

The construct validity of the UCSDSOBQ is supported by significant correlation and responsiveness between UCSDSOBQ and other anchors. Importantly, the UCSDSOBQ had stronger correlation and responsiveness with patient-reported questionnaires compared with objective PFT measures and the 6MWD, which is consistent with the findings from a previous study [9]. These data suggest that the UCSDSOBQ primarily reflects the distinct subjective aspect of dyspnea, and cannot be replaced by objective measurements. It is therefore important for clinical trials and clinicians to consider both physiological and patient-reported measurements when evaluating disease progression or response to therapy. Recent definitions of progressive fibrosing ILD include worsening dyspnea, and measures like the UCSDSOBQ may also be useful to bring some objectivity to this particular criterion for progression. As a patient-completed domain-specific questionnaire that requires minimal in-person instruction, the UCSDSOBQ is a reasonable tool to assess dyspnea. In clinical practice, the UCSDSOBQ can also vary over time partly due to extrapulmonary factors (e.g., change in mood, cardiac disease), so the upper limit of the MID (8 points) could serve as a more robust choice for clinicians who need to be more certain about disease progression. Conversely, the lower limit of the MID (5 points) is likely sufficient for testing treatment effects in large clinical trial populations.

The content validity of the UCSDSOBQ is demonstrated by the internal consistency and absence of both ceiling and floor effects, which is similar to previous analyses in COPD [30]. Internal consistency is evaluated by Cronbach’s alpha, which was 0.97 in this study and comparable to previous results ranging from 0.90 to 0.94 [15, 19, 31]. The internal consistency describes the extent to which the questions measure the same concept (i.e., dyspnea severity). The very high Cronbach’s alpha for the UCSDSOBQ suggests that this questionnaire includes redundant items and that it may be possible to eliminate some questions without a significant loss of performance. However, changing the content of the UCSDSOBQ would invalidate previous analyses describing its validity and other characteristics of the current version, and additional studies would then be needed to validate any changes to this questionnaire. The right skewing of the UCSDSOBQ in fibrotic ILD further suggests that there are likely excessive questions focused on activities that result in minimal dyspnea for a large percentage of patients, and that these specific questions should be the focus of any attempts to shorten the UCSDSOBQ.

Our study has the following limitations. First, patients were enrolled from a single country, and confirmation of these findings in other populations would be beneficial. Second, some patients lacked 6-month follow-up data at the time of analysis; however, this limitation is alleviated to a large degree by the responsiveness analysis yielding expected findings and by this analysis being based on almost 800 measurements for 6-month data and 600 measurements for 12-month data, with these patients having similar baseline features compared to the remaining patients without longitudinal data. Third, some patients in our cohort were included in the derivation of the MID for the EQ-5D-5L and EQ-VAS [6], although we obtained virtually identical results when we only used other anchors for the analysis. Fourth, our results were obtained from ILD referral centers, but the consistent findings across various subgroup analyses suggest our main findings are likely to be consistent across difference populations. Fifth, although the K-BILD is increasingly used in patients with ILD as a quality-of-life measure, this was not validated when CARE-PF was designed and we thus do not have this questionnaire available for this cohort. Finally, we did not calculate separate MIDs for improvement and worsening, primarily given the absence of robust estimates for different improvement or worsening MID values in our selected anchors. There is furthermore limited clinical relevance of a MID for improvement in dyspnea given the typically progressive natural history of fibrotic ILD and the limited potential for improvement in dyspnea with available therapies.

## Conclusion

In conclusion, this study demonstrates the validity of the UCSDSOBQ in a large and heterogeneous population of patients with fibrotic ILD and provides a robust MID estimate of 5–8 points. In most settings, a change in UCSDSOBQ of 5 points is a reasonable threshold for the MID, particularly when contextualized with other measurements (e.g., exercise limitation, pulmonary function, imaging findings). A change in UCSDSOBQ of 8 points is a more robust threshold that is more likely to be clinically meaningful even without other data to support a significant change. These findings will facilitate interpretation of previous and ongoing studies in fibrotic ILD and support the design of future clinical trials needed in this patient population.

## Supplementary Information

**Additional file 1: Table E1.** Baseline characteristics of patients with and without 6-month follow-up. **Table E2.** Baseline characteristics across ILD subtypes. **Table E3.** Change in anchors across tertiles of change in UCSDSOBQ over 12 months. **Table E4.** IPF and non-IPF subgroup analysis of anchor- and distribution-based estimates of MID for UCSDSOBQ. **Table E5.** CTD-ILD and non-CTD-ILD subgroup analysis of anchor- and distribution-based estimates of MID for UCSDSOBQ. **Table E6.** Female and male subgroup analysis of anchor- and distribution-based estimates of MID for the UCSDSOBQ. **Table E7.** Older and younger subgroup analysis of anchor- and distribution-based estimates of MID for the UCSDSOBQ.

## Data Availability

The datasets used during the current study are available from the corresponding author on reasonable request.

## Abbreviations

CARE-PF: CAnadian REgistry for Pulmonary Fibrosis
COPD: Chronic obstructive pulmonary disease
CTD: Connective tissue disease
DLCO: Diffusing capacity of the lung for carbon monoxide
EQ-5D-5L: European Quality of Life 5 Dimensions 5 Levels questionnaire
EQ-VAS: European Quality of Life visual analogue scale
FVC: Forced vital capacity
HP: Hypersensitivity pneumonitis
ILD: Interstitial lung disease
IPF: Idiopathic pulmonary fibrosis
IQR: Interquartile range
K-BILD: King's Brief Interstitial Lung Disease Questionnaire
MID: Minimum important difference
PFT: Pulmonary function test
SD: Standard deviation
SEM: Standard error measurement
SGRQ: St. George’s Respiratory Questionnaire
UCSDSOBQ: University of California, San Diego Shortness of Breath Questionnaire
6MWD: 6-Minute walk distance
