# Supplementary material for: Validation and minimum important difference of the UCSD Shortness of Breath Questionnaire in fibrotic interstitial lung disease
Source: Respir Res. 2021 Jul 8;22:202. doi: 10.1186/s12931-021-01790-0 (PMC8265065; doi:10.1186/s12931-021-01790-0)
Supplement: Supplementary file 1 — Additional file 1: Table E1. Baseline characteristics of patients with and without 6-month follow-up. Table E2. Baseline characteristics across ILD subtypes. Table E3. Change in anchors across tertiles of change in UCSDSOBQ over 12 months. Table E4. IPF and non-IPF subgroup analysis of anchor- and distribution-based estimates of MID for UCSDSOBQ. Table E5. CTD-ILD and non-CTD-ILD subgroup analysis of anchor- and distribution-based estimates of MID for UCSDSOBQ. Table E6. Female and male subgroup analysis of anchor- and distribution-based estimates of MID for the UCSDSOBQ. Table E7. Older and younger subgroup analysis of anchor- and distribution-based estimates of MID for the UCSDSOBQ. [file 12931_2021_1790_MOESM1_ESM.docx]

***Additional Appendix to:***

**Validation and minimum important difference of the
UCSD shortness of breath questionnaire in fibrotic interstitial lung disease**

*Chen et al.*

*Patient characteristics at 6 months follow up*

**Table E1.** Baseline characteristics of patients with and without 6-month follow-up.

**Table E2.** Baseline characteristics across ILD subtypes.

**Table E3.** Change in anchors across tertiles of change in UCSDSOBQ over 12 months.

**Table E4.** IPF and non-IPF subgroup analysis of anchor- and distribution-based estimates of MID for UCSDSOBQ

**Table E5.** CTD-ILD and non-CTD-ILD subgroup analysis of anchor- and distribution-based estimates of MID for UCSDSOBQ

**Table E6.** Female and male subgroup analysis of anchor- and distribution-based estimates of MID for the UCSDSOBQ.

**Table E7.** Older and younger subgroup analysis of anchor- and distribution-based estimates of MID for the UCSDSOBQ.

**Table E1.** Baseline characteristics of patients with and without 6-month follow-up.

| **Variable** | **Patients with 6-month data available** | | **Patients without 6-month data available** | |
| --- | --- | --- | --- | --- |
| Total patients | 799 | | 1134 | |
| - IPF | 220 (28%) | | 319 (28%) | |
| - CTD-ILD | 317 (40%) | | 384 (34%) | |
| - Unclassifiable ILD | 119 (15%) | | 225 (20%) | |
| - HP | 72 (9%) | | 79 (7%) | |
| - Other ILD | 71 (9%) | | 127 (11%) | |
|  |  | |  | |
| Age, years | 62 ± 12 | | 65 ± 13 | |
| Male sex | 434 (54%) | | 601 (53%) | |
| Death or transplant | 90 (11%) | | 191 (17%) | |
|  |  |  | |  |
| Ever-smoker | 496 (62%) | | 714 (63%) | |
| Smoking pack-years | 23 (7-33) | | 22 (8-39) | |
|  |  |  | |  |
| FVC, %-predicted | 73 ± 20 | | 76 ± 20 | |
| DLCO, %-predicted | 57 ± 19 | | 58 ± 20 | |
| 6MWD, meters | 457 ± 114 | | 413 ± 129 | |
|  |  |  | |  |
| SGRQ total score | 41 ± 20 | | 40 ± 22 | |
| UCSD SOBQ total score | 37 ± 27 | | 38 ± 28 | |
| EQ5D-5L | 0.80 ± 0.17 | | 0.78 ± 0.20 | |
| EQ-VAS | 70 ± 18 | | 68 ± 20 | |

Data shown are number (%), mean ± standard deviation, or median (interquartile range).

Abbreviations: 6MWD, 6-minute walk distance; CTD-ILD, connective tissue disease-associated ILD; DLCO, diffusing capacity of the lung for carbon monoxide; EQ-5D-5L, European Quality of Life 5 Dimensions 5 Levels questionnaire; EQ-VAS, European Quality of Life visual analogue scale; FVC, forced vital capacity; HP, hypersensitivity pneumonitis; ILD, interstitial lung disease; IPF, idiopathic pulmonary fibrosis; IQR, interquartile range; SD, standard deviation; SGRQ, St. George’s Respiratory Questionnaire; UCSDSOBQ, University of California, San Diego Shortness of Breath Questionnaire.

**Table E2.** Baseline characteristics across ILD subtypes.

| **Variable** | **IPF** | **CTD-ILD** | **HP** | **Unclassifiable ILD** | **Other ILDs** |
| --- | --- | --- | --- | --- | --- |
| Total sample size | 539 (28%) | 701 (36%) | 151 (8%) | 344 (18%) | 67 (3%) |
|  |  |  |  |  |  |
| Age, years | 69 ± 8 | 56 ± 13 | 61 ± 11 | 65 ± 11 | 56 ± 18 |
| Male sex | 413 (77%) | 262 (37%) | 70 (46%) | 196 (57%) | 34 (51%) |
|  |  |  |  |  |  |
| Ever-smoker | 408 (76%) | 364 (52%) | 82 (54%) | 230 (67%) | 35 (52%) |
| Smoking pack-years | 26 (11-40) | 15 (5-32) | 21 (9-36) | 23 (9-39) | 18 (7-28) |
|  |  |  |  |  |  |
| FVC, %-predicted | 76 ± 19 | 75 ± 19 | 70 ± 19 | 76 ± 20 | 78 ± 24 |
| DLCO, %-predicted | 54 ± 18 | 60 ± 20 | 56 ± 17 | 59 ± 20 | 67 ± 23 |
| 6MWD, meters | 432 ± 127 | 453 ± 128 | 409 ± 111 | 407 ± 131 | 475 ± 115 |
|  |  |  |  |  |  |
| UCSDSOBQ total score | 35 ± 26 | 36 ± 27 | 41 ± 25 | 41 ± 29 | 42 ± 30 |
| SGRQ total score | 39 ± 20 | 38 ± 22 | 46 ± 20 | 43 ± 22 | 42 ± 22 |
| EQ-5D-5L | 0.81 ± 0.17 | 0.78 ± 0.19 | 0.78 ± 0.19 | 0.77 ± 0.22 | 0.77 ± 0.20 |
| EQ-VAS | 72 ± 19 | 69 ± 19 | 66 ± 20 | 67 ± 21 | 65 ± 21 |

Data shown are number (%), mean ± standard deviation, or median (interquartile range).

Abbreviations: 6MWD, 6-minute walk distance; CTD-ILD, connective tissue disease-associated ILD; DLCO, diffusing capacity of the lung for carbon monoxide; EQ-5D-5L, European Quality of Life 5 Dimensions 5 Levels questionnaire; EQ-VAS, European Quality of Life visual analogue scale; FVC, forced vital capacity; HP, hypersensitivity pneumonitis; ILD, interstitial lung disease; IPF, idiopathic pulmonary fibrosis; SGRQ, St. George’s Respiratory Questionnaire; UCSD-SOBQ, University of California, San Diego Shortness of Breath Questionnaire.

**Table E3.** Change in anchors across tertiles of change in UCSDSOBQ over 12 months.

| **Tertiles of change in UCSDSOBQ** | **Total number of patients** | **Tertile 1** (decrease in UCSDSOBQ) | **Tertile 2**  (minimal change**)** | **Tertile 3** (increase in UCSDSOBQ) |
| --- | --- | --- | --- | --- |
| mean ∆UCSDSOBQ | 373 | -14.2 ± 14.3 | 2.0 ± 2.5 | 21.5 ± 14.5 |
| **∆ reported in SGRQ** |  | **-5.5 ± 12.7** | **-0.1 ± 8.1** | **9.0 ± 13.1** |
| mean ∆UCSDSOBQ | 555 | -12.3 ± 14.2 | 2.7 ± 2.5 | 22.1 ± 13.5 |
| **∆ reported in EQ-5D-5L** |  | **0.047 ± 0.126** | **-0.013 ± 0.105** | **-0.075 ± 0.159** |
| mean ∆UCSDSOBQ | 561 | -12.4 ± 14.1 | 2.8 ± 2.5 | 22.5 ± 14.9 |
| **∆ reported in EQ-VAS** |  | **3.3 ± 16.5** | **0.7 ± 13.9** | **-8.6 ± 19.4** |
| mean ∆UCSDSOBQ | 433 | -12.5 ± 13.7 | 2.8 ± 2.4 | 22.2 ± 15.1 |
| **∆ reported in FVC** |  | **0.7 ± 7.7** | **-0.1 ± 6.9** | **-2.9 ± 7.3** |
| mean ∆UCSDSOBQ | 336 | -12.7 ± 10.4 | 1.7 ± 2.6 | 19.3 ± 11.7 |
| **∆ reported in DLCO** |  | **-0.2 ± 8.2** | **-1.1 ± 9.2** | **-2.8 ± 9.7** |
| mean ∆UCSDSOBQ | 194 | -12.5 ± 10.4 | 1.3 ± 2.8 | 20.0 ± 12.5 |
| **∆ reported in 6MWD** |  | **11.9 ± 74.0** | **-3.4 ± 67.1** | **-42.5 ± 81.1** |

Data shown are mean ± standard deviation for the 607 patients with 12-month follow-up. This analysis included patients who had longitudinal UCSDSOBQ scores obtained 12 months after baseline. 80% of the patients in this analysis were not included in the group with 6-month follow-up data.

Abbreviations: 6MWD, 6-minute walk distance; DLCO, diffusing capacity of the lung for carbon monoxide; EQ-5D-5L, European Quality of Life 5 Dimensions 5 Levels questionnaire; EQ-VAS, European Quality of Life visual analogue scale; FVC, forced vital capacity; SGRQ, St. George’s Respiratory Questionnaire; UCSDSOBQ, University of California, San Diego Shortness of Breath Questionnaire.

**Table E4.** IPF and non-IPF subgroup analysis of anchor- and distribution-based estimates of MID for UCSDSOBQ.

| **IPF** | | | | |
| --- | --- | --- | --- | --- |
| **Anchor-based MID estimates** | | | | |
| **Anchor** | **Linear regression equation** | | **MID for anchor** | **MID for UCSDSOBQ** |
| SGRQ | UCSD = -6.51 + 1.06*SGRQ | | 5 to 8 | 5.3 to 8.5 |
| EQ-5D-5L | UCSD = 125.25 – 112.56*EQ-5D-5L | | 0.054 | 6.1 |
| EQ-VAS | UCSD = 93.15 – 0.82*EQ-VAS | | 5 | 4.1 |
| FVC% | UCSD = 73.96 – 0.52*FVC% | | 2 to 6% | 1.0 to 3.1 |
| DLCO% | UCSD = 63.21 – 0.58*DLCO% | | 11% | 6.4 |
| 6MWD | UCSD = 67.12 – 0.08*6MWD | | 20.7 to 35.4m | 1.7 to 2.8 |
| **Distribution-based MID estimates** | | | | |
| Standard error measurement approach | | 4.7 | | |
| **Non-IPF** | | | | |
| **Anchor-based MID estimates** | | | | |
| **Anchor** | **Linear regression equation** | | **MID for anchor** | **MID for UCSDSOBQ** |
| SGRQ | UCSD = -5.13 + 1.04*SGRQ | | 5 to 8 | 5.2 to 8.3 |
| EQ-5D-5L | UCSD = 112.36 – 96.55*EQ-5D-5L | | 0.054 | 5.2 |
| EQ-VAS | UCSD = 94.49 – 0.84*EQ-VAS | | 5.0 | 4.2 |
| FVC% | UCSD = 78.33 – 0.54*FVC | | 2 to 6% | 1.1 to 3.2 |
| DLCO% | UCSD = 68.14 – 0.56*DLCO | | 11% | 6.2 |
| 6MWD | UCSD = 81.34 – 0.10*6MWD | | 20.7 to 35.4m | 2.1 to 3.5 |
| **Distribution-based MID estimates** | | | | |
| Standard error measurement approach | | 4.6 | | |

Abbreviations: 6MWD, 6-minute walk distance; DLCO, diffusing capacity of the lung for carbon monoxide; EQ5D-5L, European Quality of Life 5 Dimensions 5 Levels questionnaire; EQ-VAS, European Quality of Life visual analogue scale; FVC, forced vital capacity; IPF, idiopathic pulmonary fibrosis; MID, minimum important difference; SGRQ, St. George’s Respiratory Questionnaire; UCSDSOBQ, University of California, San Diego Shortness of Breath Questionnaire.

**Table E5.** CTD-ILD and non-CTD-ILD subgroup analysis of anchor- and distribution-based estimates of MID for the UCSDSOBQ.

| **CTD-ILD** | | | |
| --- | --- | --- | --- |
| **Anchor-based MID estimates** | | | |
| **Anchor** | **Linear regression equation** | **MID for anchor** | **MID for UCSDSOBQ** |
| SGRQ | UCSD = -4.82 + 1.02*SGRQ | 5 to 8 | 5.1 to 8.2 |
| EQ-5D-5L | UCSD = 110.37 – 96.11*EQ-5D-5L | 0.054 | 5.2 |
| EQ-VAS | UCSD = 91.95 – 0.83*EQ-VAS | 5 | 4.2 |
| FVC% | UCSD = 74.46 – 0.52*FVC% | 2 to 6% | 1.0 to 3.1 |
| DLCO% | UCSD = 63.85 – 0.52*DLCO% | 11% | 5.7 |
| 6MWD | UCSD = 81.79 – 0.10*6MWD | 20.7 to 35.4m | 2.1 to 3.5 |
| **Distribution-based MID estimates** | | | |
| Standard error measurement approach | | 4.4 | |
| **non-CTD-ILD** | | | |
| **Anchor-based MID estimates** | | | |
| **Anchor** | **Linear regression equation** | **MID for anchor** | **MID for UCSDSOBQ** |
| SGRQ | UCSD = -5.93 + 1.06*SGRQ | 5 to 8 | 5.3 to 8.5 |
| EQ-5D-5L | UCSD = 117.87 – 102.42*EQ-5D-5L | 0.054 | 5.5 |
| EQ-VAS | UCSD = 95.36 – 0.84*EQ-VAS | 5 | 4.2 |
| FVC% | UCSD = 78.88 – 0.55*FVC | 2 to 6% | 1.1 to 3.3 |
| DLCO% | UCSD = 66.86 – 0.56*DLCO | 11% | 6.2 |
| 6MWD | UCSD = 75.52 – 0.10*6MWD | 20.7 to 35.4m | 2.1 to 3.5 |
| **Distribution-based MID estimates** | | | |
| Standard error measurement approach | | 4.5 | |

Abbreviations: 6MWD, 6-minute walk distance; CTD-ILD, connective tissue disease-associated interstitial lung disease; DLCO, diffusing capacity of the lung for carbon monoxide; EQ-5D-5L, European Quality of Life-5 Dimensions 5 Levels questionnaire; EQ-VAS, European Quality of Life visual analogue scale; FVC, forced vital capacity; MID, minimum important difference; SGRQ, St. George’s Respiratory Questionnaire; UCSD-SOBQ, University of California, San Diego Shortness of Breath Questionnaire.

**Table E6.** Female and male subgroup analysis of anchor- and distribution-based estimates of MID for the UCSDSOBQ.

| **Male** | | | |
| --- | --- | --- | --- |
| **Anchor-based MID estimates** | | | |
| **Anchor** | **Linear regression equation** | **MID for anchor** | **MID for UCSDSOBQ** |
| SGRQ | UCSD = -5.25 + 1.03*SGRQ | 5 to 8 | 5.2 to 8.2 |
| EQ-5D-5L | UCSD = 114.23 – 99.76*EQ-5D-5L | 0.054 | 5.4 |
| EQ-VAS | UCSD = 92.46 – 0.83*EQ-VAS | 5 | 4.2 |
| FVC% | UCSD = 74.14 – 0.52*FVC | 2 to 6% | 1.0 to 3.1 |
| DLCO% | UCSD = 64.12 – 0.55*DLCO | 11% | 6.1 |
| 6MWD | UCSD = 72.27 – 0.09*6MWD | 20.7 to 35.4m | 1.9 to 3.2 |
| **Distribution-based MID estimates** | | | |
| Standard error measurement approach | | 4.3 | |
| **Female** | | | |
| **Anchor-based MID estimates** | | | |
| **Anchor** | **Linear regression equation** | **MID for anchor** | **MID for UCSDSOBQ** |
| SGRQ | UCSD = -5.74 + 1.06*SGRQ | 5 to 8 | 5.3 to 8.5 |
| EQ-5D-5L | UCSD = 115.97 –100.19*EQ-5D-5L | 0.054 | 5.4 |
| EQ-VAS | UCSD = 95.96 – 0.84*EQ-VAS | 5 | 4.2 |
| FVC% | UCSD = 81.67 – 0.57*FVC% | 2 to 6% | 1.1 to 3.4 |
| DLCO% | UCSD = 69.53 – 0.57*DLCO% | 11% | 6.3 |
| 6MWD | UCSD = 83.56 – 0.11*6MWD | 20.7 to 35.4m | 2.3 to 3.9 |
| **Distribution-based MID estimates** | | | |
| Standard error measurement approach | | 4.6 | |

Abbreviations: 6MWD, 6-minute walk distance; DLCO, diffusing capacity of the lung for carbon monoxide; EQ-5D-5L, European Quality of Life-5 Dimensions 5 Levels questionnaire; EQ-VAS, European Quality of Life visual analogue scale; FVC, forced vital capacity; MID, minimum important difference; SGRQ, St. George’s Respiratory Questionnaire; UCSD-SOBQ, University of California, San Diego Shortness of Breath Questionnaire.

**Table E7.** Older and younger subgroup analysis of anchor- and distribution-based estimates of MID for the UCSDSOBQ.

| **Younger: Age ≤ 65 years** | | | |
| --- | --- | --- | --- |
| **Anchor-based MID estimates** | | | |
| **Anchor** | **Linear regression equation** | **MID for anchor** | **MID for UCSDSOBQ** |
| SGRQ | UCSD = -4.6 + 0.99*SGRQ | 5 to 8 | 5.0 to 7.9 |
| EQ-5D-5L | UCSD = 111.02 – 96.37*EQ-5D-5L | 0.054 | 5.2 |
| EQ-VAS | UCSD = 93.05 – 0.83*EQ-VAS | 5 | 4.2 |
| FVC% | UCSD = 76.49 – 0.54*FVC% | 2 to 6% | 1.1 to 3.2 |
| DLCO% | UCSD = 65.85 – 0.52*DLCO% | 11% | 5.7 |
| 6MWD | UCSD = 82.18 – 0.10*6MWD | 20.7 to 35.4m | 2.1 to 3.5 |
| **Distribution-based MID estimates** | | | |
| Standard error measurement approach | | 4.4 | |
| **Older: Age > 65 years** | | | |
| **Anchor-based MID estimates** | | | |
| **Anchor** | **Linear regression equation** | **MID for anchor** | **MID for UCSDSOBQ** |
| SGRQ | UCSD = -7.42 + 1.14*SGRQ | 5 to 8 | 5.7 to 9.1 |
| EQ-5D-5L | UCSD = 121.14 – 105.82*EQ-5D-5L | 0.054 | 5.7 |
| EQ-VAS | UCSD = 95.40 – 0.85*EQ-VAS | 5 | 4.3 |
| FVC% | UCSD = 79.05 – 0.55*FVC | 2 to 6% | 1.1 to 3.3 |
| DLCO% | UCSD = 66.57 – 0.59*DLCO | 11% | 6.5 |
| 6MWD | UCSD = 79.87 – 0.12*6MWD | 20.7 to 35.4m | 2.5 to 4.2 |
| **Distribution-based MID estimates** | | | |
| Standard error measurement approach | | 4.6 | |

Abbreviations: 6MWD, 6-minute walk distance; DLCO, diffusing capacity of the lung for carbon monoxide; EQ-5D-5L, European Quality of Life-5 Dimensions 5 Levels questionnaire; EQ-VAS, European Quality of Life visual analogue scale; FVC, forced vital capacity; MID, minimum important difference; SGRQ, St. George’s Respiratory Questionnaire; UCSD-SOBQ, University of California, San Diego Shortness of Breath Questionnaire.
